# Peer Review of “Modeling Years of Life Lost Due to COVID-19, Socioeconomic Status, and Nonpharmaceutical Interventions: Development of a Prediction Model”

**DOI:** 10.2196/37985

**Published:** 2022-04-12

**Authors:** Bozhidar Chakalov

**Affiliations:** 1 University of California, Davis Davis, CA United States

**Keywords:** COVID-19, pandemic, socioeconomic status, mortality, nonpharmaceutical interventions, prediction model, low-income status, life expectancy, public health, income groups

*This is a peer-review report submitted for the paper “Modeling Years of Life Lost Due to COVID-19, Socioeconomic Status, and Nonpharmaceutical Interventions: Development of a Prediction Model.”*

## Review Round 1

### General Comments

The paper [1] is very well written and is very timely. I believe that this model will be informative in that it shows how long-term solutions rather than short term are needed to avoid greater losses in the long term. However, as the authors say, the model is based on somewhat weaker empirical research, so I look forward to seeing this model validated with data.

### Specific Comments

#### Major Comments

1. I think it should be made clearer that this model is applied to US and European scenarios.

2. I believe that the paper and research are well motivated and show a necessity for more research on the proportional impact of socioeconomic status on years of life lost.

#### Minor Comments

3. There are several cases where the authors should correct some typos (eg, the European [the European what?] and sill vs still).
